# Frequency And Factors Associated With Adverse Reactions After Administration of Inactivated COVID-19 Vaccine Among Health Workers

**DOI:** 10.1590/0037-8682-0152-2023

**Published:** 2023-09-22

**Authors:** Beray Gelmez Taş, İlknur Demir, Muhammed Basanmay, Güzin Zeren Öztürk, Bestegül Çoruh Akyol, Merve Hicret Tektaş, Hacı Mustafa Özdemir

**Affiliations:** 1 University of Health Sciences, Sisli Hamidiye Etfal Research and Training Hospital, Department of Family Medicine, Istanbul, Turkey.; 2 Ordu University, Faculty of Medicine, Department of Family Medicine, Ordu, Turkey.; 3 Sapanca Public Hospital, Department of Family Medicine, Sakarya, Turkey.; 4 University of Health Sciences, Sisli Hamidiye Etfal Research and Training Hospital, Department of Orthopedics and Traumatology, Istanbul, Turkey.

**Keywords:** COVID-19 vaccines, Adverse reactions, CoronaVac vaccine

## Abstract

**Background:**

The 2019 coronavirus (COVID-19) has precipitated a significant public health crisis. Our study aimed to evaluate the prevalence and risk factors associated with adverse reactions to the inactivated CoronaVac vaccine.

**Methods:**

The study involved voluntary health workers who received CoronaVac vaccine. We documented the sociodemographic information of 2,019 participants who volunteered for our study. Of these, 1,964 and 1,702 participants were interviewed by phone 1 month after the first and second dose, respectively, during which they were queried about any adverse reactions.

**Results:**

Within the first week after the first dose, adverse reactions were observed in 856 (43.3%) participants, with 133 (6.7%) experiencing them during the second week, and 96 (4.9%) people at the end of the first month. For the second dose, 276 individuals (16.2%) reported adverse reactions. The prevalence of both local and systemic adverse events ranged from 9.5-11.2% overall. Fatigue was the most common adverse reaction overall, while pain at the injection site was the most frequent local adverse reaction.

**Conclusions:**

The evaluation of both systemic and local side effects revealed no significant adverse reactions to the inactivated CoronaVac vaccine (Sinovac Life Sciences, Beijing, China). Our study found that the incidence of systemic and local adverse responses to the CoronaVac vaccination was lower than the rates reported in studies involving the recombinant adenovirus type-5, BNT162b1, and ChAdOx1nCoV-19 COVID-19 vaccines, all of which underwent the World Health Organization LULUC/PQ evaluation process.

## INTRODUCTION

Coronavirus disease 2019 (COVID-19) was first discovered in Wuhan, China, and the outbreak, which began on December 12, 2019, rapidly expanded around the globe^1^​. As there is no proven antiviral therapy for COVID-19, the primary focus remains on the development of coronavirus vaccines^2^. Throughout the pandemic, countries worldwide have implemented various measures to curb the disease’s spread. Although Turkey has not yet reported any COVID-19 cases, the Ministry of Health established a scientific committee in January 2020 in response to the situation in China^3^. The first guidelines were published online on January 14, 2020, and numerous scientific programs were conducted either online or through in-service training^3^. Several measures, including a curfew for individuals aged ≤20 and those aged ≥65 have been implemented to prevent transmission^4^ ^,^ ^5^.

The COVID-19 infection caused a global public health issue and economic crisis, prompting the rapid development of vaccines and therapeutic measures^1^. Vaccines harness the immune system’s ability to recognize and recall encounters with pathogenic antigens^6^. A vaccine emerged as a crucial solution to the COVID-19 pandemic, which proliferated rapidly and exhibited exceedingly high mortality rates.

Typically, the development of a new vaccine requires a span of 10 to 15 years^7^​. The mumps vaccine, however, set a record by being created and approved for use in approximately 5 years^7^​. Therefore, there is a significant work underway to develop a vaccine against COVID-19​^8^. Adenovirus vector vaccines, RNA vaccines, protein subunit vaccines, DNA plasmid vaccines, inactivated vaccinations, and virus-like particle vaccines are a few examples of candidate vaccines currently undergoing clinical testing. Currently, there are 183 candidate vaccines in clinical examination and 199 candidate vaccines in preclinical evaluation for COVID-19, according to the World Health Organization (WHO). Among the ongoing clinical vaccine studies, 12% (22) involve inactivated vaccines^9^.

With vaccine-induced neutralizing antibodies, CoronaVac, an inactivated COVID-19 vaccine developed by Sinovac Life Sciences, Beijing, China, demonstrates robust immunogenicity. It is capable of inducing vaccine-induced neutralizing antibodies, effectively neutralizing severe acute respiratory syndrome coronavirus (SARS-CoV-2) in mice, rats, and non-human primates^10^. In April 2021, WHO published the interim/topline efficacy results (CoronaVac phase 3) of Evidence Assessment of Sinovac/CoronaVac COVID-19 vaccine. The results indicated that CoronaVac exhibited efficacy rates of 51% in Brazil, 84% in Turkey, and 65% in Indonesia against symptomatic COVID-19 cases. The data evaluated by the WHO suggests that the benefits of Sinovac-CoronaVac outweigh any known or potential disadvantages^11^.

Although a study involving participants from many countries revealed these findings, it is noteworthy that vaccine hesitancy was higher among individuals with greater concerns about potential side-effects^12^. Hence, a critical factor influencing vaccination rates is the occurrence of vaccine adverse reactions, which can lead to reservations about vaccination.

Administration of the vaccine named “CoronaVac 600 SU/0.5 ml IM Injection Suspension Containing Vial” to health workers in Turkey was initiated on January 14, 2021, marking the commencement of the nationwide COVID-19 vaccination process​^13^. This study was conducted to evaluate the prevalence and risk factors of adverse reactions to the recently approved inactivated CoronaVac vaccine (Sinovac Life Sciences, Beijing, China), marking its inaugural use in Turkey.

## METHODS

The study, conducted between February 5, 2021, and April 15, 2021, at the Health Sciences University Şişli Hamiye Etfal Training and Research Hospital Health Practice Research Center, involved voluntary health workers who were vaccinated with CoronaVac (Sinovac Life Sciences, Beijing, China). Health workers received two doses of 3 μg/0.5 mL (equivalent to 600 SU per doe) intramuscular (deltoid) vaccine, with a 28-day interval between doses.

Before conducting the research, ethical approval was acquired from the Health Sciences University's Ethics Committee at the Şişli Hamidiye Etfal Health Training and Research Hospital, with reference number 3123, on February 2, 2021. All participants provided both verbal and written consent prior to their involvement in the study. 

Vaccination of health workers in our hospital began on January 14, 2021. Following ethical permission, the vaccine recipients were identified retrospectively. Those who chose to participate in the study engaged in face-to-face interviews. Sociodemographic data (age, sex, occupation, year of employment, and department) and communication data were obtained. They were contacted by phone and queried about any adverse reactions (both local and systemic) during the first week, second week, first month, and one month after the second dose. 

The sociodemographic characteristics of 2,019 people who consented to participate in our study were recorded. Of these, 1,964 and 1,702 were contacted 1 month after the first and second dose, respectively, and their adverse reactions were investigated. Total 317 individuals were excluded from the study owing to inadequate communication, voluntary withdrawal from the study, and not receiving the second dose as a result of testing positive for PCR.

IBM SPSS version 25.0, provided by SPSS Inc., Chicago, Illinois, USA, was utilized for all statistical analyses. Continuous variables are represented as Mean [standard deviation (SD)] in the tables. Certain categorical variables are tabulated, while others are denoted as numbers (N) and percentages (%). The chi-square test was employed to compare categorical variables. Multivariate logistic regression analysis was used to identify variables predicting negative reactions. A significance level of 5% was established. 

## RESULTS

In our study, 2,019 individuals consented to participate, and we documented their sociodemographic characteristics. We managed to contact 1,964 and 1,702 of these participants 1 month after their first and second dose, respectively, to investigate any adverse reactions. The average age of the participants was 35.54 years with an SD of 10.79, and 15.3% (n=260) had contracted COVID-19 prior to vaccination. Table 1 presents the sociodemographic data of the participants who completed the study.1

**TABLE 1:** Sociodemographic Data of the Participants (n=1,702).

Demographic variables	n or X_ort_ (min-max)	% or Mean[SD]
**Sex**		
Female	970	57.0
Male	732	43.0
**Age (year)**	32.0 (20.0-67.0)	35.54[10.79]
20-30 years	793	46.6
31-40 years	369	21.7
41-50 years	342	20.1
Above 50 years	198	11.6
**Job title**		
Doctor	536	31.5
Nurse	450	26.4
Cleaning staff	43	2.5
Other	673	39.5
**Branch (unit)**		
Policlinic	456	26.8
Clinic	952	55.9
Administrative units	172	10.1
Other	122	7.2
**Alcohol abuse**		
No	1,291	75.9
Yes	411	24.1
**Smoking**		
No	1,113	65.4
Yes	589	34.6
**Chronic disease**		
No	1,428	83.9
Yes	274	16.1
**Allergy**		
No	1,445	84.9
Yes	257	15.1
**How many times did you have URTI in a year before the COVID-19 period?**		
<5	1,644	96.6
5-10	51	3.0
>10	7	0.4
**Have you had COVID-19 before?**		
No	1,442	84.7
Yes	260	15.3
**Is there a risk-group individual at home? (n=1889)**		
**a. Pregnant**		
No	1,379	86.0
Yes	224	14.0
**b. Child**		
No	1,326	82.7
Yes	277	17.3
**c. Above 65 years**		
No	1,276	79.6
	327	20.4

**FIGURE 1:**
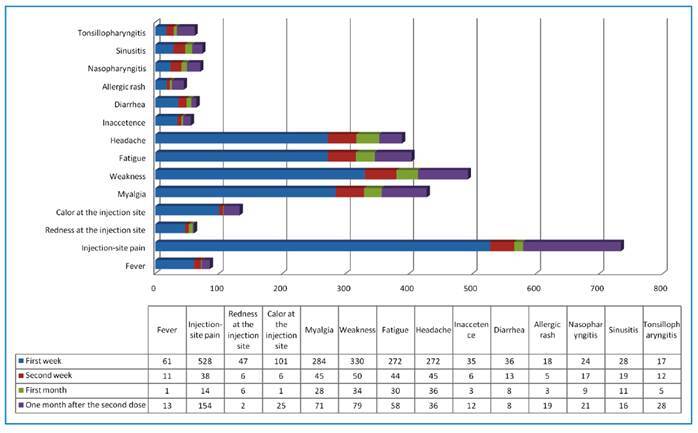
Distribution of adverse reactions to vaccination in the first week, second week, and first month after the first dose and one month after the second dose.

Adverse reactions were observed in 856 (43.3%) people 1 week after the first dose; in 133 (6.7%) people, in the second week; and in 96 (4.9%) people, at the end of the first month; 276 (16.2%) individuals experienced adverse reactions 1 month after the second dose. No serious adverse reactions were reported. Types of adverse reactions are presented in Figure 1. 

The most frequent adverse reaction was pain at the injection site, followed by fatigue as the most prevalent systemic reaction was weariness. Pain at the injection site was also the most frequent local adverse reaction. The frequency distribution of systemic and local adverse reactions is shown in Table 2. 

**TABLE 2:** Frequency distribution of systemic and local adverse reactions of the participants.

Adverse Reaction Status	First week	Second week	First month	One month after the second dose
	n=1,975	n=1,976	n=1,964	n=1,704
No adverse reaction	1,119 (56.7)	1,823 (92.3)	1,868 (95.1)	1,428 (83.8)
Only systemic adverse reaction	297 (15.0)	109 (5.5)	13 (0.7)	114 (6.7)
Only local adverse reaction	236 (11.9)	20 (1.0)	77 (3.9)	85 (5.0)
Systemic + local adverse reaction	323 (16.4)	24 (1.2)	6 (0.3)	77 (4.5)

Table 3 presents a comparison of certain sociodemographic variables, with and without adverse reactions, following the second dose. A statistically significant difference was observed in response to the question, “Did you experience any side effects at the end of the second month?" This difference was significant in relation to sex (p<0.001), chronic disease (p=0.025), and the question, “Did you experience any side effects at the end of the first month?” (p<0.001). 

**TABLE 3:** Comparison of second dose with and without adverse reactions and sociodemographic variables.

	Second Dose With and Without Adverse Reactions			
Variables	Groups	No (n=1,426)	Yes (n=276)	p-value
Age	20-30 years	658 (83)	135 (17)	0.232
	31-40 years	312 (84.6)	57 (15.4)	
	41-50 years	281 (82.2)	61 (17.8)	
	Above 50 years	175 (88.4)	23 (11.6)	
Sex	Female	776 (80)	194 (20)	**<0.001**
	Male	650 (88.8)	82 (11.2)	
Job title	Doctor	443 (82.6)	93 (17.4)	0.366
	Nurse	371 (82.4)	79 (17.6)	
	Cleaning staff	35 (81.4)	8 (18.6)	
	Other	577 (85.7)	96 (14.3)	
Branch (unit)	Policlinic	384 (84.2)	72 (15.8)	0.676
	Clinic	162 (83)	952 (17)	
	Administrative units	26 (84.9)	172 (15.1)	
	Other	16 (86.9)	122 (13.1)	
Alcohol abuse	No	1,075 (83.3)	216 (16.7)	0.307
	Yes	351 (85.4)	60 (14.6)	
Smoking	No	928 (83.4)	185 (16.6)	0.533
	Yes	498 (84.6)	91 (15.4)	
Chronic disease	No	1,209 (84.7)	219 (15.3)	**0.025**
	Yes	217 (79.2)	57 (20.8)	
Allergy	No	1,215 (84.1)	230 (15.9)	0.427
	Yes	211 (82.1)	46 (17.9)	
URTI	<5	1,379 (83.9)	265 (16.1)	0.794
	5-10	41 (80.4)	10 (19.6)	
	>10	6 (85.7)	1 (14.3)	
Have you had COVID-19 before?	No	1,206 (83.6)	236 (16.4)	0.693
	Yes	220 (84.6)	40 (15.4)	
	Yes	275 (84.1)	52 (15.9)	
Have you experienced any adverse reactions in the first month?	No	810 (89.6)	94 (10.4)	**<0.001**
	Yes	581 (77.1)	173 (22.9)	

Pearson Chi-Square test.

The regression equation reveals the significance levels of the variables used in the model. Vaccine adverse reactions (first month), age (OR=0.98, 95% CI 0.97-0.99), and female sex (OR=2.39, 95% CI 1.97-2.89) were significantly related. These findings are presented in Table 4. In Model 1, it was established that approximately 7% of the factors determining vaccine adverse reactions (first month) could be explained by the variables of decreasing age and female sex.

**TABLE 4:** Results of multivariate logistic regression on having adverse reactions to vaccination 1 month after the first and second dose.

	Vaccine adverse reaction one month after the first dose (Model 1)		Vaccine adverse reaction one month after the second dose (Model 2)	
Variables	OR (95%CI)	p-value	OR (95%CI)	p-value
Age	0.98 (0.97-0.99)	<0.001*	0.99 (0.97-1.00)	0.154
Sex	2.37 (1.95-2.87)	<0.001**	1.91 (1.43-2.55)	<0.001**
Smoking	1.14 (0.93-1,39)	0.193	1.07 (0.80-1.43)	0.639
Alcohol abuse	0.98 (0.78-1.23)	0.918	0.86 (0.62-1.20)	0.384
Chronic disease	1.10 (0.84-1.44)	0.473	1.43 (1.01-2.04)	0.043***
Allergy	1.22 (0.95-1.59)	0.117	0.98 (0.68-1.42)	0.934
Influenza vaccine	1.303 (0.80-1.32)	0.793	1.30 (0.94-1.81)	0.108
Pneumococcal vaccine	0.96 (0.67-1.38)	0.847	1.22 (0.77-1.93)	0.391
Undergo COVID-19	0.94 (0.73-1.21)	0.644	0.93 (0.64-1.34)	0.710

*decreasing age, **being female, ***having a chronic disease.

Among the variables used in the model, experiencing adverse reactions (second month), female sex (OR=1.91,95% CI 1.43-2.55), and having a chronic disease (OR=1.43, 95% CI 1.01-2.04) exhibited significantly associations. The variables of being female and having a chronic disease accounted for approximately 3% of the factors contributing to vaccine adverse reactions (second month) in Model 1. 

## DISCUSSION

Infectious diseases contribute to a higher number of premature deaths compared other health conditions, posing a substantial threat to public health. Deeper understanding of pathogens have initiated a transformative shift, resulting in the explanation and management of historical illnesses such as smallpox, polio, and measles over the last 150 years. However, in recent decades, a consistent upsurge in newly identified infections has captured increasing attention^14^.

Vaccines represent the traditional method of establishing a long-lasting immune memory for the management of infectious diseases. Recent advances in technology have facilitated the faster development of vaccines than ever before^9^.

Careful preclinical and clinical research are essential for designing a vaccination that ensures both safety and efficacy, minimizing significant adverse reactions^15^. The global imperative for a coronavirus vaccine underscores the need for meticulous benefit-risk assessments to inform medical and legal decisions, thereby identifying potential risks and challenges^9^.

Ensuring vaccine safety is paramount. Studies have shown that a 6 μg dose of CoronaVac was protective in macaque monkeys, with no adverse effects on mental state, appetite, or other bodily functions^10^. Moreover, when these vaccinated macaques were exposed to SARS-CoV-2, they exhibited protection against the virus, as evidenced by low viral loads, unlike the control group^10^. A review assessing the decrease in symptomatic COVID-19 incidence compared to a placebo revealed varying levels of vaccine efficacy (VE): low certainty evidence for CoronaVac (Sinovac) (VE 69.81%, 95% CI 12.27%-89.61%; 2 RCTs, 19,852 participants), and high certainty of evidence for the following VEs: BNT162b2: 97.84%, 95% CI 44.25%-99.92%; 2 RCTs, 44,077 participants; mRNA-1273: 93.20%, 95% CI 91.06%-94.83%; 2 RCTs, 31,632 participants; ChAdOx1: 70.23%, 95% CI 62.10-76.62%; 2 RCT, 43,390 participants; Ad26.COV2.S: 66.90%, 95% CI 59.10%-73.40%, 1 RCT, 39,058 participants; BBIBP-CorV: 78.10%, 95% CI 64.80%-86.30%; 1 RCT, 25,463 participants; BBV152: 77.80%, 95% CI 65.20%-86.40%; 1 RCT, 16,973 participants^6^.

A separate study revealed that the antibody response rate was 20% after administering a single dose of CoronaVac. However, following two doses, the vaccine's efficacy increased to 90.3%^16^. Our research focused on evaluating the adverse reactions to the inactivated CoronaVac vaccine (Sinovac Life Sciences, Beijing, China). No fatal adverse reactions were observed during our follow-up. Adverse reactions were reported by 856 (43.3%) out of 1,964 individuals contacted 1 week after the first dose and by 276 (16.2%) out of 1, 702 individuals contacted 1 month after the second dose. Our findings align with the CoronaVac Phase 2 study, which reported an overall incidence rate of adverse events^17^ at 15.0 %. In another study, 22 healthcare workers who had received the CoronaVac vaccine and experienced at least one adverse event were included^16^. The rates are comparable, and the variations may be attributed to the fact that individual reactions to vaccination, similar to SARS-CoV-2 infection immunity^18^, can differ.

Our research, similar to a previous study (67.9%), found that female healthcare workers experienced adverse reactions more frequently (78.28%)^19^. Among the viral vaccines that caused more side effects in women than in men were the flu, MMR vaccine, attenuated Japanese encephalitis, and attenuated dengue vaccine trials^20^. The disparity in adverse reactions by sex may be attributed to theories related to adaptive immunity, sex steroid-related theories, and theories associated with innate immunity^20^. We posit that further research is necessary to ascertain why adverse reactions are more prevalent in women. In our study, the overall incidence of local and systemic adverse events ranged from 9.5%-11.2%%. This was significantly lower than the rates reported for other SARS-CoV-2 vaccine platform candidates^21^ ^-^ ^26^ and more comparable to the rates of other inactivated SARS-CoV-2 vaccine candidates^17^ ^,^ ^24^.

Localized pain at the injection site was the most common adverse reaction in our trial, which was similar to that observed in the CoronaVac Phase 2 study (n=154; 9.0%). Fatigue and muscle soreness were the most prevalent systemic adverse reactions (n=79; 4.6% each). All adverse reactions were mild to moderate. The vaccine was well-tolerated, and no serious vaccine-related adverse reactions were observed. Another study found that localized pain or itching at the injection site was the most common systemic adverse event, with incidence rates of 9.6% after the first dose and 10.7% after the second dose​^27^. Both trials' adverse event reporting profiles matched those of previously reported phase 3 clinical studies^28^ ^-^ ^30^.

In a study examining individuals who sought emergency department care post-receipt of the Coronovac vaccine, fatigue was the most frequently reported symptom, with a prevalence of 29.7%. Upon evaluation in the emergency department, the most prevalent diagnoses were upper respiratory infection (28.6%) and myalgia (32.1%). Notably, none of the patients required ventilator support or hospitalization, and all were discharged^31^.

With the progression of research and the passage of time post-vaccination, rare adverse reactions such as deafness, acute urticaria, papulopustular eruption, lichenoid eruption, and herpes zoster have been increasingly reported^2^ ^,^ ^32^ ^,^ ^33^. We believe that meticulous recording and analysis of adverse reactions should be sustained over many years. This is crucial to ensure the safe administration of vaccines and to mitigate vaccine hesitancy.

## CONCLUSION

No major adverse reactions to the inactivated CoronaVac vaccine (Sinovac Life Sciences, Beijing, China) have been documented after evaluation for both systemic and local side effects. The incidence of systemic and local adverse responses to the CoronaVac vaccination in our study was lower than those reported in studies using the recombinant adenovirus type-5 and BNT162b1, ChAdOx1nCoV-19 COVID-19 vaccines that underwent the WHO LULUC/PQ evaluation process. However, rare adverse reactions are still being reported. Therefore, more studies focusing on real-life adverse reaction are warranted.

## References

[1] Acar H, Gökseven Y, Öztürk GZ, Arıca S (2020). Covid-19 In Primary Healthcare. Ankara Med J.

[2] Zhao H, Li Y, Wang Z (2022). Adverse event of Sinovac Coronavirus vaccine: Deafness. Vaccine.

[3] Taş BG, Özceylan G, Öztürk GZ, Toprak D (2021). Evaluation of Job Strain of Family Physicians in COVID-19 Pandemic Period- An Example from Turkey. J Community Health.

[4] Taş BG, Selvi HR, Öztürk GZ, Arıca SG, Egici MT (2022). Evaluation of Knowledge, Practices, and Attitudes Towards Coronavirus in Individuals Aged 20-64 Years. Hamidiye Med J.

[5] (2023). Curfew Circular for 65 Years and Over and Those with Chronic Disorders.

[6] Graña C, Ghosn L, Evrenoglou T, Jarde A, Minozzi S, Bergman H (2022). Eicacy and safety of COVID-19 vaccines (Review). Cochrane Database Syst Rev.

[7] Sharma O, Sultan AA, Ding H, Triggle CR (2019). A Review of the Progress and Challenges of Developing a Vaccine for COVID-19. Front Immunol.

[8] WHO (2023). COVID-19 vaccines.

[9] (2023). Sinovac: CoronaVac - COVID19 Vaccine Tracker.

[10] Gao Q, Bao L, Mao H, Wang L, Xu K, Yang M Development of an Inactivated Vaccine Candidate for SARS-CoV-2.

[11] Wilder-Smith A, Hombach J, Marti M, Desai S, O’Brien K (2022). Interim recommendations for use of the inactivated COVID-19 vaccine, CoronaVac, developed by Sinovac. WHO.

[12] Lu JG (2022). Two large-scale global studies on COVID-19 vaccine hesitancy over time: Culture, uncertainty avoidance, and vaccine side-effect concerns. J Pers Soc Psychol.

[13] Health Republic of Turkey Ministry of (2023). COVID-19 Vaccine National Implementation Strategy.

[14] Graham BS, Sullivan NJ (2018). Emerging viral diseases from a vaccinology perspective: Preparing for the next pandemic review-article. Nat Immunol.

[15] Graham BS (2020). Rapid COVID-19 vaccine development. Science.

[16] Eyupoglu G, Guven R, Karabulut N, Cakir A, Sener K, Genc Yavuz B (2023). Humoral responses to the CoronoVac vaccine in healthcare workers. Rev Soc Bras Med Trop.

[17] Xia S, Duan K, Zhang Y, Zhao D, Zhang H, Xie Z (2020). Effect of an Inactivated Vaccine Against SARS-CoV-2 on Safety and Immunogenicity Outcomes: Interim Analysis of 2 Randomized Clinical Trials. J Am Med Assoc.

[18] Poland GA, Ovsyannikova IG, Kennedy RB (2020). SARS-CoV-2 immunity: review and applications to phase 3 vaccine candidates. Lancet.

[19] Riad A, Sa D, Gıro ˘ Glu ˘, Üstün B, Pokorná A, Klugarová J (2021). Prevalence and Risk Factors of CoronaVac Side Effects: An Independent Cross-Sectional Study among Healthcare Workers in Turkey. J Clin Med.

[20] Klein SL, Jedlicka A, Pekosz A (2010). The Xs and Y of immune responses to viral vaccines. Lancet Infect Dis.

[21] Mulligan MJ, Lyke KE, Kitchin N, Absalon J, Gurtman A, Lockhart S (2020). Phase I/II study of COVID-19 RNA vaccine BNT162b1 in adults. Nature.

[22] Walsh EE, Frenck RW, Falsey AR, Kitchin N, Absalon J, Gurtman A (2020). mRNA Vaccine against SARS-CoV-2 - Preliminary Report. N Engl J Med.

[23] Zhang Y, Zeng G, Pan H, Li C, Hu Y, Chu K (2020). Safety and immunogenicity of the ChAdOx1 nCoV-19 vaccine against SARS-CoV-2: a preliminary report of a phase 1/2, single-blind, randomised controlled trial. Lancet.

[24] Zhang M-X, Zhang T-T, Shi G-F, Cheng F-M, Zheng Y-M, Tung T-H (2021). Safety, tolerability, and immunogenicity of an inactivated SARS-CoV-2 vaccine in healthy adults aged 18-59 years: a randomised, double-blind, placebo-controlled, phase 1/2 clinical trial. Lancet Infect Dis.

[25] Tanriover MD, Doğanay HL, Akova M, Güner HR, Azap A, Akhan S (2020). Immunogenicity and safety of a recombinant adenovirus type-5-vectored COVID-19 vaccine in healthy adults aged 18 years or older: a randomised, double-blind, placebo-controlled, phase 2 trial. Lancet.

[26] Palacios R, Batista AP, Albuquerque CSN, Patiño EG, Santos J do P, Tilli Reis Pessoa Conde M (2020). Safety and Immunogenicity of Two RNA-Based Covid-19 Vaccine Candidates. N Engl J Med.

[27] Zhang MX, Zhang TT, Shi GF, Cheng FM, Zheng YM, Tung TH (2021). Safety of an inactivated SARS-CoV-2 vaccine among healthcare workers in China. Expert Rev Vaccines.

[28] Tanriover MD, Doğanay HL, Akova M, Tanrıöver MD, Doğanay HL, Akova M, Güner HR, Azap A, Akhan S (2021). Efficacy and safety of an inactivated whole-virion SARS-CoV-2 vaccine (CoronaVac): interim results of a double-blind, randomised, placebo-controlled, phase 3 trial in Turkey. Lancet.

[29] Palacios R, Patiño EG, de Oliveira Piorelli R, Conde MTRP, Batista AP, Zeng G (2020). Double-Blind, Randomized, Placebo-Controlled Phase III Clinical Trial to Evaluate the Efficacy and Safety of treating Healthcare Professionals with the Adsorbed COVID-19 (Inactivated) Vaccine Manufactured by Sinovac - Profiscov: A structured summary of a. Trials.

[30] Palacios R, Batista AP, Albuquerque CSN, Patiño EG, Santos JP, Conde MTRP (2021). Efficacy and Safety of a COVID-19 Inactivated Vaccine in Healthcare Professionals in Brazil: The PROFISCOV Study. SSRN Electron J.

[31] Semih Gedik M, Kilci Aİ, Hakkoymaz H, Faruk Küçük Ö, Solak Y, Mehmet Basan N (2023). Evaluation of patients of vaccine side effects after the COVID-19 vaccine. Rev Assoc Med Bras.

[32] Öner Ü, Aktaş A (2023). Cutaneous reactions after CoronaVac and BioNTech vaccines. J Cosmet Dermatol.

[33] Aksu SB, Öztürk GZ (2021). A rare case of shingles after COVID-19 vaccine: is it a possible adverse effect?. Clınıcal Experımental Vaccıne Res.

